# Chemical composition and biological activity of lemongrass volatile oil and *n*-Hexane extract: GC/MS analysis, *in vitro* and molecular modelling studies

**DOI:** 10.1371/journal.pone.0319147

**Published:** 2025-02-25

**Authors:** Shaza H. Aly, Abdullahi Ibrahim Uba, Nilofar Nilofar, Taghreed A. Majrashi, Mahmoud A. El Hassab, Wagdy M. Eldehna, Gokhan Zengin, Omayma A. Eldahshan

**Affiliations:** 1 Department of Pharmacognosy, Faculty of Pharmacy, Badr University in Cairo (BUC), Cairo, Egypt,; 2 Department of Molecular Biology and Genetics, Istanbul AREL University, Istanbul, Turkey,; 3 Department of Biology, Science Faculty, Selcuk University, Konya, Turkey,; 4 Department of Pharmacy, Botanic Garden “Giardino dei Semplici”, Università degli Studi “Gabriele d’Annunzio”, via dei Vestini 31, Chieti, Italy,; 5 Department of Pharmacognosy, College of Pharmacy, King Khalid University, Asir, Saudi Arabia,; 6 Department of Medicinal Chemistry, Faculty of Pharmacy, King Salman International University (KSIU), South Sinai, Egypt,; 7 Department of Pharmaceutical of Chemistry, Faculty of Pharmacy, Kafrelsheikh University, Kafrelsheikh, Egypt,; 8 Department of Pharmaceutical Chemistry, Faculty of Pharmacy, Pharos University in Alexandria, Canal El Mahmoudia St., Alexandria, Egypt,; 9 Pharmacognosy Department, Faculty of Pharmacy, Ain Shams University, Cairo, Egypt; Cukurova University: Cukurova Universitesi, TÜRKIYE

## Abstract

Lemon grass, formally identified as *Cymbopogon citratus*, is a plant that belongs to the Poaceae family. The present work aimed to examine the chemical composition by GC/MS analysis and assess the biological potential of *C. citratus* volatile oil and *n*-hexane extract. The volatile oil and *n*-hexane extract were evaluated for antioxidant potential and tested for their enzyme inhibition against tyrosinase, butyrylcholinesterase (BChE), acetylcholinesterase (AChE), *α*-amylase, and *α*-glucosidase. The chemical analysis of the lemongrass n-hexane extract (HE) and volatile oil (VO) revealed that the main constituents in the HE are aliphatic hydrocarbons (42.98%), triterpenoids (20.14%), and aromatic hydrocarbons (17.25%). Conversely, the main constituents of the (VO) are predominantly monoterpenes, namely α-citral (36.08%), *β*-citral (34.22%), and *β*-myrcene (13.84%). The oil showed more potent antioxidant potential in DPPH, ABTS, CUPRAC, FRAP, and phosphomolybdenum (10.18, 35.69 mg Trolox equivalent/g, 98.97 and 69.73 mg Trolox equivalent/g and 43.01 mmol Trolox equivalent/g). The HE displayed higher BChE (1.53 mg Galanthamine equivalent)/g), as well as *α*-amylase and *α*-glucosidase inhibitory activities (0.39 and 2.40 mmol Acarbose equivalent/g). The VO demonstrated more potent tyrosinase inhibitory activities (57.19 mg Kojic acid equivalent/g) along with acetyl and butyrylcholinesterase inhibition. Dominant compounds exhibited the ability to bind with high affinity to various target proteins, with a particular affinity for AChE and BChE. The volatile oil and *n*-hexane extract of *C. citratus* show significant promise as a viable choice for the advancement of novel therapeutic strategies aimed at addressing oxidative stress, neurodegeneration, and diabetes.

## 1. Introduction

In public and primary healthcare, medicinal plants have evolved into the most prominent and safest alternative medications [1,2]. These therapeutic herbs are essential agents within the primary healthcare system, contributing to the overall well-being and maintenance of good health [3–5]. Notably, more than two-thirds of the world’s population currently depends on medicinal plants as a fundamental source of therapeutic medications [6]. The rise in popularity of these natural therapies can be ascribed to their ubiquitous acceptability, compatibility, flexibility, and the negligible negative effects encountered when applied to the human body [7–9].

*Cymbopogon* is a genus within the Poaceae (Gramineae) family, comprising approximately 144 identified species. Its distribution spreads extensively across tropical and subtropical areas of Africa, Asia, and Central America. The abundance of essential oils in this plant has earned it a reputation for its widespread usage in fragrance, skin care products, and therapeutic applications [10]. One of the most renowned species in the *Cymbopogon* genus is *C. citratus*, typically known as Lemongrass and found worldwide [10]. It is well known for its lemon-like essence owing to the presence of citral as its major component [11]. Lemongrass remains a popular traditional remedy for addressing the plethora of ailments such as coughs, epithelial diseases, flu, pneumonia, malaria, headaches, gingivitis, leprosy, and vascular disorders. Additionally, in certain regions, it is employed to alleviate acne, pimples, and blackheads, as well as to combat lice and dandruff [11,12].

Lemongrass is a highly abundant reservoir of several secondary metabolites, including the volatile oil that constitutes citral, as well as polyphenolic components such as luteolin, apigenin, chlorogenic acid, *p*-coumaric acid, and caffeic acid [13–15]. Notably, monoterpenes are the main constituents of the hydrodistilled oil that is obtained from *Cymbopogon* species [16]. In addition, several pharmacological investigations on *C. citratus* have demonstrated its medicinal potential as an antioxidant, antibacterial, antiaging, hypoglycemic, cytotoxic, anti-inflammatory, and insect-repellent agent [17–20]. Various reports on the biological activity of various *Cymbopogon* species have been published [12]. For example, Rhimi et al. reported the antifungal activity of *C. citratus* and *C. proximus* known as (Halfbar) towards *Candida* spp [16]. The *C. citratus* methanolic extract exhibited antioxidant and α-glucosidase inhibitory properties [20]. Another investigation provided evidence that the ethyl extract of *C. citratus* is efficacious in the management of acne vulgaris [11]. *Cymbopogon citratus* is distinguished from other Cymbopogon species by its unique fragrant characteristics, diverse food and therapeutic uses, abundant phytochemical profile, and considerable economic significance [10,21].

Molecular docking was selected as the optimal computational technique to evaluate the underlying mechanism of inhibitory effect for the biologically active constituents, thereby elucidating the interactions between the enzyme and the principal secondary metabolites [22–24]. It involves predicting the preferred orientation and binding affinity of small molecules (ligands) to target proteins or receptors, which is essential for understanding molecular interactions [25]. The primary purpose of the present research is to provide insight into the differences in the phytochemical composition of the volatile oil and *n*-hexane extract of *C. citratus* by GC/MS analysis as the method of analysis. Additionally, various *in vitro* studies have been conducted to systematically evaluate the potential of *C. citratus* volatile constituents as antioxidant, anti-Alzheimer’s, anti-diabetic, and anti-tyrosinase agents. Molecular modeling was conducted to investigate the interactions between the tested enzymes and major constituents. These findings present compelling opportunities for the potential use of *C. citratus* in managing various illnesses. The graphical abstract summarizes the chemical analysis, and the *in vitro* biological assays performed on *Cymbopogon citratus* volatile oil.

## 2. Materials and methods

### 2.1. Plant Material

The fresh leaves of lemon grass (*Cymbopogon citratus*) were acquired in February 2022 from a privately-owned farm placed in Shibin Al Kawm City, Al Minufiyah, Egypt. Professor Usama K. Abdel Hameed from the Department of Botany, Faculty of Science, Ain Shams University, Cairo, Egypt, verified the authenticity of the plant. The Pharmacognosy Department, Faculty of Pharmacy, Ain Shams University, Cairo, Egypt received a voucher specimen for the deposit, identified by the code PHG-P-CC-463.

### 2.2. Isolation of the volatile oil of lemongrass

Finely chopped fresh leaves weighing 500 gm of *C. citratus* were hydrodistilled using distilled water for 5 hours on a Clevenger apparatus. Upon completion of the distillation process, the light, yellow-coloured essential oil was gathered, measured, and dehydrated using anhydrous sodium sulphate. The mean hydrodistillation output was 0.25% (v/w). The sample was stored in sealed opaque glass vials at a temperature of -4 °C until it was subjected to GC/MS and subsequent examination.

### 2.3. Preparation of the n-hexane extract of lemongrass

In the extraction procedure, 100 gm of desiccated leaves of *C. citratus* were subjected to cold maceration in distilled analytical quality *n*-hexane (Nasr Pharmaceuticals, Cairo, Egypt) for 48 hours until complete exhaustion. After filtering and concentrating the extracts in vacuo at 45 °C until dry, the dried residue (3.1 g) was obtained with a yield of 3.1%. The yield was determined per 100 gm of lemon grass and reported as a percentage (w/w) [26]. For further investigation, the extract was kept in a tightly sealed container in the refrigeration unit.

### 2.4. Gas chromatography/mass spectrometry (GC/MS) analysis

GC/MS analysis was performed on a Kyoto, Japan-made Shimadzu GCMS-QP 2010 chromatograph with a DB-5 capillary column from Bellefonte, PA, USA. It has a 30 m length, 0.25 mm internal diameter, and 0.25 µm film thickness. The injection temperature was 250 °C. After 2 min of isothermal setup at 45 °C, the temperatures were raised to 300 °C at 5 °C/min and kept isothermal for 5 min. Helium was used as the inert carrier gas at 1.41 mL/min. Diluted samples (1% volume/volume) were injected at a 15:1 split ratio with a 1 µ L injection volume. The study was conducted using the following MS operating parameters: the interface temperature was kept constant at 280 °C, the ion-source temperature was set at 220 °C, the electron ionisation (EI) mode was employed at 70 eV, and the scan range extended from 35 to 500 amu. The hexane sample was exposed to the same conditions, except the oven temperature. More precisely, the oven temperature was first adjusted to 50 °C and maintained for 3 minutes. Next, the temperature was raised at an average rate of 5 °C per minute until it reached 300 °C and then held at that level for 10 minutes [27–29]. To identify the essential oil components, the mass fragmentation data (MS) and Kovats indices (KI) were compared with the data found in the NIST Mass Spectral Library (2011) and the 4^th^ edition of Adams [30] with the information provided in the 8^th^ edition of the Wiley Registry of Mass Spectral Data, together with relevant details documented in the scientific literature [31–34].

### 2.5. Total phenolic and flavonoid content

To determine the total phenolic and flavonoid contents, the Folin-Ciocalteu and AlCl_3_ tests were employed [35]. The findings of the studies were reported as gallic acid equivalents (mg GAEs/g sample) and rutin equivalents (mg REs/g sample). All relevant experimental information can be found in the Supplementary Materials.

### 2.6. Antioxidant assays

The antioxidant activity of the samples under investigation was evaluated in triplicate using well-established techniques as previously described [36,37]. Positive controls in the antioxidant studies included trolox and EDTA. Quantification of the radical scavenging activity of DPPH and ABTS, together with CUPRAC and FRAP, was performed and reported as mg of Trolox equivalents (TE) per gram of the measured sample. The metal chelating assay (MCA) quantified the amount of ethylenediaminetetraacetic acid equivalents (EDTAE) per gram of the evaluated sample. Total antioxidant activity, measured by the phosphomolybdenum test (PM), was expressed as mmol TE/g extract [38,39].

### 2.7. Enzyme inhibition assays

An analysis was conducted to examine the inhibitory properties of the essential oils against AChE, BChE, tyrosinase, amylase, and glucosidase. Previous methodologies were employed to ascertain the activities in triplicate [36,37]. AChE and BChE inhibitory activities were quantified in mg of galanthamine equivalents (GALAE)/ g of the tested sample, tyrosinase in mg of kojic acid equivalents (KAE)/g of the tested sample, and amylase and glucosidase in mg of acarbose equivalents (ACAE)/g of the tested sample [38,39].

### 2.8. Molecular modeling

The X-ray crystal structures of the specifically chosen target proteins were obtained from the protein data bank available at (https://www.rcsb.org/) [40] α-amylase (PDB ID: 1B2Y) [41], AChE (PDB ID: 6O52) [42], BChE (PDB ID: 6EQP) [43]. A novel homology model of human tyrosinase was constructed by utilizing natural sequences (UniProt entries: P14679) and the three-D structure of tyrosinase from Priestia megaterium (PDB ID: 6QXD) [44].; and that of α-glucosidase was constructed using the 3D structure of Mus musculus glucosidase (PDB ID: 7KBJ) [45] as a template [46] from human sequence (UniProt entries: P0DUB6). All proteins have been created following the previously detailed procedure [47]. The 3D structures of the ligands were derived from the ChemSpider database (https://www.chemspider.com/) and then optimized using the UCSF Chimaera software [48]. Every docking grid file was generated using the cocrystal ligand of each protein using MGLTools 1.5.6 software. This software combined all hydrogen atoms and assigned gasteiger partial charges to all protein atoms. Computer simulation of docking was conducted using the Lamarckian genetic process in AutoDock 4.2.6 software available at https://autodock.scripts.edu [49] and adopting the docking protocol [50]. Binding energy scores for ligand poses were calculated and protein-ligand interaction was investigated using Biovia DS Visualizer v4.5 from BIOVIA in San Diego, CA, USA.

### 2.9. Statistical analysis

The experiments were performed in triplicate, and differences between the volatile oil and hexane extract were compared using the Multiple t-test (*p* < 0.05). GraphPad Prism 6.01 (GraphPad Inc., La Jolla, CA, USA).

## 3. Results and discussion

### 3.1. GC/MS analysis of the volatile oil and n-hexane extract of lemongrass

**Fig 1:**
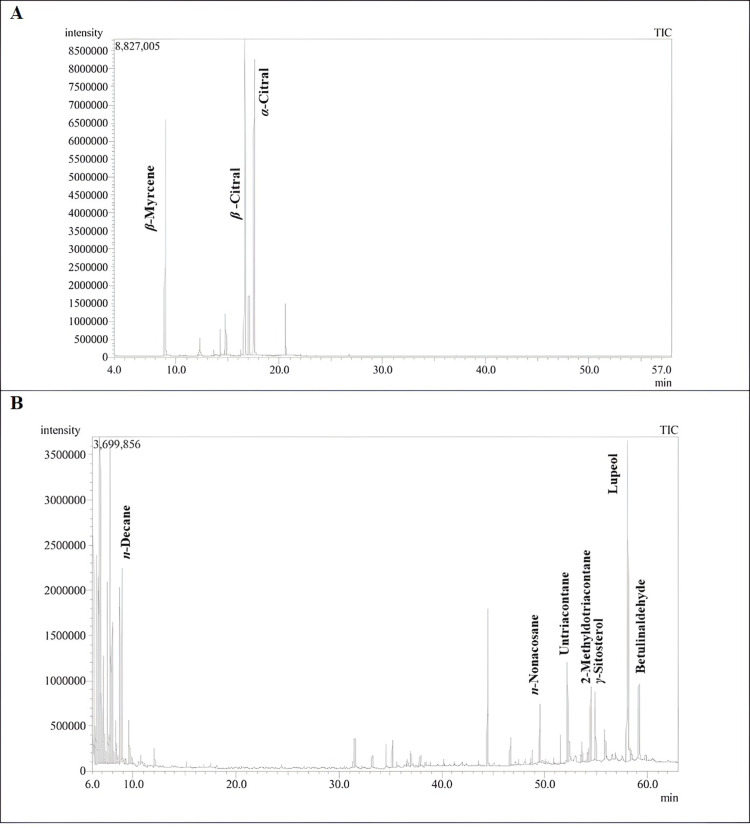
GC chromatogram of (A) Volatile oil and (B) *n*-Hexane extract of Lemongrass.

**Fig 2:**
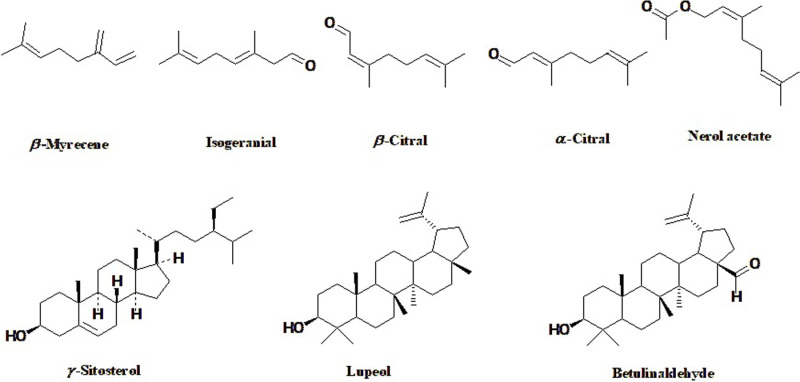
Chemical structures of the major constituents identified in the volatile and *n*-hexane extract of Lemongrass using GC/MS analysis.

The abundance of bioactive phytochemicals found in plants is primarily responsible for their potential biological properties [4,8,9,51–53]. In the current study, The GC/MS analysis was employed for both qualitative and quantitative analysis of the constituents in the volatile oil of the lemongrass and *n*-hexane extract (Fig 1). Table 1 provides the identified compounds, their percentages, and the retention indices. A total of fifty-three and twelve compounds were identified in the VO and HE of lemon grass accounting for 100% and 87.56%, respectively. On one hand, the VO was predominated with the oxygenated monoterpenes (84.95%) followed by monoterpene hydrocarbons (13.84%). Where the major constituents were found to be *α*-citral (36.08%), *β* -citral (34.22%) and *β*-myrcene (13.84%). On the other hand, the HE was predominated with aliphatic hydrocarbons (42.98%), triterpenoids (20.14%), and aromatic hydrocarbons (17.25%). Where, lupeol 16.23%) and betulinaldehyde (3.91%). Regarding the hydrocarbons, *n*-propylcyclohexane, octane, 2,6-dimethyl, 3-cyclohexylpropyl alcohol, hemellitol, and *n*-decane were the major identified components in the hexane extract. The chemical structures of the major identified constituents are represented in (Fig 2).

**Table 1:** Chemical composition (%) of the volatile oil and *n*-hexane extract of lemongrass using GC/MS analysis.

No.	Rt _(min)_	Compound	RI_Exp_.^a^	RI_Lit_^b^	Molecular Formula	Content (%)	
						HE	VO
1	6.08	1-Ethyl-4-methylcyclohexane	901	896	C_9_H_18_	2.82	**–**
2	6.16	Cyclohexane, 1-ethyl-1-methyl-	903	903	C_9_H_18_	1.32	**–**
3	6.43	**3-Cyclohexylpropyl alcohol**	912	–	C_9_H_18_O	**5.40**	**–**
4	6.60	**2-Phenylpropane**	918	919	C_9_H_12_	**3.65**	**–**
5	6.73	***n*-Propylcyclohexane**	923	924	C_9_H_18_	**6.27**	**–**
6	6.85	**Octane, 2,6-dimethyl**	927	933	C_10_H_22_	**4.26**	**–**
7	7.06	Heptane, 3-ethyl-2-methyl	934	940	C_10_H_22_	1.98	**–**
8	7.19	2,6-Dimethyloctane	938	935	C_10_H_22_	0.74	**–**
9	7.32	2,4,4,6-Tetramethyl-2-heptene	943	942	C_11_H_22_	0.25	**–**
10	7.70	*m*-Ethyltoluene	956	958	C_9_H_12_	6.19	**–**
11	7.76	Octane, 4,5-diethyl-	958	–	C_12_H_26_	3.49	**–**
12	7.91	Mesitylene; 1,3,5-Trimethylbenzene	963	964	C_9_H_12_	2.87	**–**
13	8.26	*o*-Ethylmethylbenzene	975	973	C_9_H_12_	0.83	**–**
14	8.40	*trans*-*p*-Menthane 1-Methyl-4-(1-methyl ethyl)-cyclohexane	979	978	C_10_H_20_	0.47	**–**
15	8.48	*cis*-Hydrindan	982	981	C_9_H_16_	0.17	**–**
16	8.68	**Hemellitol**	989	996	C_9_H_12_	**3.71**	**–**
17	8.79	Sulcatone (Methyl heptanone)	987	987	C_8_H_14_O	**–**	1.21
18	8.88	***n*-Decane**	996	1000	C_10_H_22_	**3.48**	**–**
19	8.94	***β*-Myrcene**	992	992	C_10_H_16_	**–**	**13.84**
20	9.21	2,5-Dimethylnonane	1007	1024	C_11_H_24_	0.10	**–**
21	9.58	5-Ethyl-2-methyl heptane	1018	1019	C_10_H_22_	0.96	**–**
22	9.85	Butylcyclohexane	1027	1030	C_10_H_20_	0.10	**–**
23	10.69	Isobutyl acetoacetate	1053	1060	C_8_H_14_O_3_	0.13	**–**
24	12.03	*n*-Undecane	1096	1100	C_11_H_24_	0.48	**–**
25	12.26	*β*-Linalool	1099	1099	C_10_H_18_O	–	1.09
26	13.66	7-Methyl-3-methyleneoct-6-enal	1145	1146	C_10_H_16_O	**–**	0.33
27	14.26	Isoneral	1165	1183	C_10_H_16_O	**–**	1.59
28	14.60	Rose furan oxide	1176	1174	C_10_H_14_O_2_	**–**	0.34
29	14.81	Isogeranial	1183	1184	C_10_H_16_O	**–**	2.27
30	16.26	Nerol	1231	1228	C_10_H_18_O	**–**	0.53
31	16.67	***β* -Citral (Neral)**	1246	1248	C_10_H_16_O	**–**	**34.22**
32	17.05	*trans*-Geraniol (Lemonol)	1259	1259	C_10_H_18_O	**–**	5.65
33	17.57	***α*-Citral (Geranial)**	1276	1277	C_10_H_16_O	**–**	**36.08**
34	20.58	Nerol acetate	1384	1381	C_12_H_20_O_2_	**–**	2.85
35	31.36	Neophytadiene	1825	1830	C_20_H_38_	0.11	**–**
36	31.51	Hexahydrofarnesyl acetone	1832	1835	C_18_H_36_O	0.62	**–**
37	33.20	Palmitic acid, methyl ester	1920	1923	C_17_H_34_O_2_	0.25	**–**
38	34.57	Palmitic acid, ethyl ester	1987	1990	C_18_H_36_O_2_	0.45	**–**
39	35.16	Isopropyl palmitate	2017	2023	C_19_H_38_O_2_	0.59	**–**
40	36.54	Linoleic acid, methyl ester	2088	2082	C_19_H_34_O_2_	0.12	**–**
41	36.68	Linolenic acid, methyl ester	2096	2096	C_19_H_32_O_2_	0.19	**–**
42	36.96	Stearic acid, methyl ester	2110	2109	C_19_H_38_O_2_	0.52	**–**
43	37.69	Linoleic acid	2150	2152	C_18_H_32_O_2_	0.11	**–**
44	37.79	Linoleic acid ethyl ester	2156	2155	C_20_H_36_O_2_	0.21	**–**
45	37.93	Linolenic acid, ethyl ester	2163	2169	C_20_H_34_O_2_	0.30	**–**
46	40.20	*n*-Tricosane	2289	2300	C_23_H_48_	0.13	**–**
47	46.63	*n-*Heptacosane	2688	2700	C_27_H_56_	0.61	**–**
48	47.43	Palmitic acid, neryl ester	2742	2726	C_26_H_48_O_2_	0.12	**–**
49	48.60	Squalene	2823	2823	C_30_H_50_	0.14	**–**
50	49.51	*n*-Nonacosane	2887	2900	C_29_H_60_	1.22	**–**
51	50.88	*n*-Triacontane	2986	3000	C_30_H_62_	0.11	**–**
52	51.49	*n*-Octacosanal	3032	3032	C_28_H_56_O	0.66	**–**
53	52.21	Untriacontane	3087	3100	C_31_H_64_	2.21	**–**
54	52.35	Octacosanol	3098	3110	C_28_H_58_O	0.94	**–**
55	53.51	Dotriacontane	3187	3200	C_32_H_66_	0.21	**–**
56	53.62	*β*-Sitosterol	3195	3197	C_29_H_50_O	0.54	**–**
57	54.08	Stigmasterol	3227	3248	C_29_H_48_O	0.27	**–**
58	54.20	Triacontanal	3236	3251	C_30_H_60_O	0.32	**–**
59	54.45	2-Methyldotriacontane	3253	3259	C_33_H_68_	2.36	**–**
60	54.93	*n*-Tritriacontane	3286	3300	C_33_H_68_	1.91	**–**
61	55.82	*γ*-Sitosterol	3342	3351	C_29_H_50_O	1.63	**–**
62	58.07	**Lupeol**	3554	3450	C_30_H_50_O	**16.23**	**–**
63	58.30	*γ*-Sitostenone	3570	3458	C_29_H_48_O	0.55	**–**
64	58.45	*n*-Hexatriacontane	3581	3600	C_36_H_74_	0.35	**–**
65	59.14	**Betulinaldehyde**	3629	3628	C_30_H_48_O_2_	**3.91**	**–**
**Monoterpene Hydrocarbons**						**0.47**	**13.84**
**Oxygenated Monoterpenes**						**–**	**84.95**
**Diterpenoids**						**0.11**	**–**
**Sesquiterpene Hydrocarbon**						**0.14**	**–**
**Oxygenated Sesquiterpene**						**0.62**	**–**
**Triterpenoids**						**20.14**	**–**
**Sterols**						**2.99**	**–**
**Fatty acids and fatty acids derivatives**						**2.86**	**–**
**Aromatic Hydrocarbons**						**17.25**	**–**
**Aliphatic Hydrocarbons**						**42.98**	**–**
**Others**						**–**	**1.21**
**Total identified compounds**						**87.56**	**100**

^a^Retention index calculated experimentally on DB-5 column relative to n-alkane series (C8–C28).

^b^Reported retention indices. Compounds are listed in order of their elution on DB-5 GC column.

Prior investigations on the essential oil constituents of *C. citratus* leaves have shown that the primary recognizable constituents are found to be the two isomers of citral and *β*-myrcene [54]. Moustafa et al. showed that *C. citratus* leaves from El-Kanater El-Khairiya, Egypt, comprise the main oil components as *α*-citral and *β*-citral, accounting for 35.91% and 35% respectively [55]. Additionally, the oil of *C. citratus* leaves from Eastern Nepal showed a similar pattern, with neral (36.1%) and geranial (53.1%) monoterpene aldehydes as the predominant components, and smaller quantities of (*E*)-caryophyllene and caryophyllene oxide [56]. While the roots are rich in α-elemol, geranial, neral and *α*-eudesmol [56]. Meanwhile, the *n*-hexane extract of *C. citratus* stem and rhizomes from Malaysia predominated with fatty acids as linoleic acid, methyl palmitate, 7,10-octadecadienoic acid, methyl ester, in addition to stigmasterol [57]. According to another research conducted by Cortes-Torres et al., the essential oil from the leaves of *C. citratus* obtained from Puebla, Mexico revealed a significant presence of myrcene, *Z*-geranial, and *E*-geranial [58]. This work conducted by Guerrini et al. focuses on the detection of volatile components in cultivated plants of *C. citratus* growing in an Amazonian Ecuador region using two different approaches; the first method involved hydrodistillation of the oil followed by GC/MS analysis, which indicates that the primary constituents were oxygenated monoterpenes (89.05%), with geraniol, geranial, and neral being the most abundant. On the other hand, the headspace fractions analysis revealed a lower percentage of oxygenated monoterpenes (68.64%), with *cis*-Isocitral and *trans*-Isocitral being the major components [59]. Another study revealed nine components in *C. citratus* oil and eight in *C. proximus*. The main compounds in *C. citratus* were geranial and neral, while *C. proximus* had piperitone and α-terpinolene [16]. Based on the aforementioned findings, it can be deduced that citral, specifically neral and geranial, is consistently found in high concentrations in *C. citratus* from various sources and various geographical locations. Therefore, citral can serve as a crucial indicator for the identification and verification of *C. citratus* which was characterized in the hydrodistilled oil. Significantly, the chemical composition of the volatile oil obtained through hydrodistillation differs from that of the hexane extract, as the hydrodistillation method is more adept at extracting a greater variety of volatile compounds, particularly lighter and more volatile components [26]. Hexane extraction mainly extracts non-polar compounds, including lipids, fats, and hydrophobic compounds such as terpenes and certain aromatic compounds [27,29].

### 3.2. Total phenolic and flavonoid content of n-hexane extract of lemongrass

The increasing prevalence of natural antioxidants, particularly polyphenols, can be ascribed to their advantageous impact on the health of humans. Polyphenols possess the capacity to alleviate the oxidative stress sequences linked to several diseases including cancer, diabetes, inflammation, wound healing, cardiovascular abnormalities, and neurodegenerative disorders [60–64]. Total phenolic and flavonoid content in the (HE) of lemongrass leaves was quantitatively determined. The findings showed high phenolic levels represented as 7.44 ±  0.34 mg GAE/g (gallic acid equivalent) and total flavonoid content of 1.91 ±  0.06 mg RE/g (rutin equivalent). The presence of phenolics in the hexane extract would add value to its antioxidant activity and mitigate various illnesses associated with oxidative stress such as neurodegeneration disorders [65,66]. In prior research on the total flavonoids and phenolics in various lemongrass extracts, a previous study on the ethanol extract of lemongrass leaves showed TPC equivalent to 67.28 mg GAE/g extract [67]. In another study, using the aqueous extract it showed TPC and TFC equivalent to 10.7 ±  0.2 mg GAE/g and 23.9 ±  0.3 mg quercetin/g, respectively [68]. Also, the concentrations of total phenolics and flavonoids in the ethyl acetate extract were 132.31 mg caffeic acid/g extract and 104.50 mg naringin/g extract, respectively [11].

### 3.3. The in vitro antioxidant potential of volatile oil and n-hexane extract of lemongrass

The present work used many assays to assess the in vitro antioxidant properties of the VO and HE of Lemongrass. By the phosphomolybdenum test, the total antioxidant capacity of the VO was determined to be 43.01 ±  0.06 mmol TE/g. Besides, the VO was found to possess notable reducing activity as evidenced by its activity in the CUPRAC and FRAP assays (98.97 ±  1.12 and 69.73 ±  1.10 mg TE/g, respectively). Similarly, it demonstrated notable radical scavenging potential in both the DPPH and ABTS assays (10.18 ±  0.43 and 35.69 ±  0.31 mg TE/g, respectively). It is noteworthy that the HE radical scavenging potential in the DPPH experiment exhibited a similar pattern, measuring 6.86 ±  0.81 mg TE/g. It was notable that the VO and HE of lemongrass showed a significantly different metal chelating activity of 31.16 ±  0.88 and 25.76 ±  0.56 mg EDTAE/g, respectively. Whereby the HE demonstrated lower reducing potential in CUPRAC and FRAP assays (30.57 ±  3.44 and 12.50 ±  0.14 mg TE/g, respectively). Moreover, the HE has a lower total antioxidant capacity of 1.00 ±  0.04 mmol TE/g revealed by the phosphomolybdenum assay (Table 2).

**Table 2:** Antioxidant potential of the volatile oil and *n*-hexane extract isolated from the Lemongrass.

Samples	DPPH	ABTS	CUPRAC	FRAP	MCA	PM
	(mg TE/g)	(mg TE/g)	(mg TE/g)	(mg TE/g)	(mg EDTAE/g)	(mmol TE/g)
**Volatile oil**	10.18 ± 0.43^a^	35.69 ± 0.31^a^	98.97 ± 1.12^a^	69.73 ± 1.10^a^	31.16 ± 0.88^a^	43.01 ± 0.06^a^
***n*-Hexane extract**	6.86 ± 0.81^b^	5.97 ± 0.33^b^	30.57 ± 3.44^b^	12.50 ± 0.14^b^	25.76 ± 0.56^b^	1.00 ± 0.04^b^

Values expressed as means ±  S.D. of three parallel measurements. TE: Trolox equivalent; EDTAE: Ethylenediaminetetraacetic acid equivalent; n.a.: not active. Different letters indicate significant differences in the extracts (*p < *0.05).

The volatile oil has shown relatively greater antioxidant activity in scavenging and reducing tests. The observed outcome can be ascribed to the high levels of bioactive constituents found in the VO as compared to the HE (Table 1). The presence of the major compounds; *α*-citral, *β*-citral and *β*-myrcene accounts for the volatile oil’s possible antioxidant action. The findings of our study were consistent with those of prior investigations, the essential oil of lemongrass at a concentration of 100 µ L/mL in ethanol showed DPPH inhibition by 76.30 ±  1.23% [58]. Interestingly, different plants with high percentages of oxygenated monoterpenes represented in *α*-citral and *β*-citral showed antioxidant potential. A previous study on *Cinnamomum bodinieri* revealed the high amount of citral isomers and recorded a high antioxidant activity with IC_50_ values of 6.887 ±  0.151 and 19.08 ±  0.02 mg/mL in DPPH and ABTS assays, respectively [58]. A recent study by Guerrini et al. in an Amazonian region of Ecuador examined the antioxidant potency of cultivated plants of *C. citratus*, the results revealed IC_50_ values of 2.270 ±  0.340 and 4.322 ±  0.651 mg/mL in DPPH and ABTS assays, respectively [59]. The petroleum ether fraction of lemon grass showed antioxidant activity by 0.12 ±  0.0082 and 0.042 ±  0.0045 mmol TE/g in DPPH and FRAP assays, respectively [11]. Regarding the major identified constituents, citral showed antioxidant due to the DPPH scavenging activity and hepatoprotective properties [69,70]. Gupta et al. showed that lupeol elevated the antioxidant levels and reduced the level of thiobarbituric acid-reactive oxygen species [71]. Also, lupeol showed reduction in the reactive oxygen species in the neurons acting as therapeutic agent in neurodegenerative disorders [72]. Moreover, lupeol isolated from *Ficus pseudopalma* exhibited antioxidant activity in DPPH and FRAP assays [73].

### 3.4. Enzyme inhibitory potential of the volatile oil and n-hexane extract of lemongrass

The application of enzyme inhibitors has important consequences in the management and treatment of diseases [74]. Consequently, extensive research efforts have been and continue to be undertaken to explore novel enzyme inhibitors that can enhance the effectiveness of therapies administered to patients. To examine the volatile oil and *n*-hexane extract efficiency as enzyme inhibitors, four important enzymes (acetyl-/butyryl-cholinesterase, tyrosinase, *α*-amylase, and *α*-glucosidase) were used. (Table 3) illustrates the findings.

**Table 3:** Enzyme inhibitory effects of the volatile oil and *n*-hexane extract isolated from the Lemongrass.

Samples	AChE Inhibition	BChE Inhibition	Tyrosinase Inhibition	*α*-Amylase Inhibition	*α*-Glucosidase Inhibition
	(mg GALAE/g)	(mg GALAE/g)	(mg KAE/g)	(mmol ACAE/g)	(mmol ACAE/g)
**Volatile oil**	1.45 ± 0.17	1.01 ± 0.23^a^	57.19 ± 5.87^a^	n.a.	n.a.
***n*-Hexane extract**	n.a.	1.53 ± 0.08^a^	45.71 ± 0.80^a^	0.39 ± 0.003	2.40 ± 0.005

Values expressed as means ±  S.D. of three parallel measurements. GALAE: Galanthamine equivalent; KAE: Kojic acid equivalent; ACAE: Acarbose equivalent; n.a.: not active. Similar letters indicate non-significant differences in the extracts (*p < *0.05).

The VO exhibited inhibitory effects on three examined enzymes: AChE, BChE, and tyrosinase, with values of 1.45 ±  0.17 mg GALAE/g, 1.01 ±  0.23 mg GALAE/g, and 57.19 ±  5.87 mg KAE/g, respectively. While the HE displayed BChE and tyrosinase inhibitory activities of 1.53 ±  0.08 mg GALAE/g and 45.71 ±  0.80 mg KAE/g, respectively. Moreover, the HE showed antidiabetic activity represented in inhibition of *α*-amylase and *α*-glucosidase equivalent to 0.39 ±  0.003 and 2.40 ±  0.005 mmol ACAE/g, respectively) by contrast the VO did not display any *α*-amylase or *α*-glucosidase inhibitory activities.The lack of inhibitory activity in the VO compared to the significant effects observed with the HE can be explained by differences in chemical composition, nature of inhibition, and potential synergistic effects among compounds present in each extract. The presence of lupeol as a major compound in the hexane extract, postponed carbohydrate digestion and absorption, thereby diminishing postprandial hyperglycaemia in rats as well as inhibiting α-amylase enzyme [75].

The enzyme acetylcholinesterase operates rapidly to degrade acetylcholine, a neurotransmitter, therefore terminating transmission between neurons at cholinergic synapses. Enzyme inhibitors are included in the therapeutic regimen for several neurological disorders and are presently the principal medication authorised by the FDA for the management of Alzheimer’s disease (AD) [76,77]. Naturally occurring cholinesterase inhibitors, specifically from plant source, have gained significant interest in this regard, as they have shown effectiveness in the management of Alzheimer’s disease (AD) [78,79]. A prior investigation conducted by Madi et al. demonstrated that the essential oil of *C. citratus* exhibits acetylcholinesterase inhibitory activity in various seasons. Specifically, the oil sample in winter demonstrated moderate AchE inhibitory activity with an IC_50_ value of 2.86 ±  0.17 mg/mL, which was comparatively to physostigmine (IC_50_ 0.012 mg/mL) [54]. The regulation of blood sugar to control hyperglycaemia in diabetic patients is based on the inhibition of carbohydrate-metabolizing enzymes; *α*-amylase and *α*-glucosidase [80]. The observed antidiabetic findings align with prior research on the petroleum extract that showed an IC_50_ value of 1.77 ±  0.55 *µ *g/mL towards *α* -glucosidase [11]. A previous study found that the methanol extract of *C. citratus* inhibits *α*-amylase with an IC_50_ value of 0.31 mg/mL. At a concentration of 1 mg/mL, the hexane extract inhibited *α*-glucosidase by 99% [81]. The promising tyrosinase inhibitory activity of lemongrass oil following previous reports regarding the potential effects of *n*-hexane extract of lemongrass on tyrosinase, elastase and collagenase enzymes with inhibitory percentages of 25.41 ±  0.68 and 49.05 ±  1.20, 72.89 ±  1.97%, respectively [11]. The interactions between catalytic residues and Cu² ⁺ ions in tyrosinase are fundamental to its enzymatic function, influencing substrate binding, catalysis, and regulatory mechanisms [82]. The volatile constituents can indeed function as catalytic residues in various enzymatic reactions due to their ability to donate or accept electrons, participate in redox reactions, and stabilize reaction intermediates [83]. Citral the major constituent in lemongrass oil showed a non-competitive inhibitory effect against a fungal source of tyrosinase [84]. Lupeol showed *in vitro* anti-tyrosinase activity along with other skin-related enzymes such as elastase [85]. Regarding the cholinesterase activity, citral from *Cymbopogon flexuosus* showed AChE inhibitory activity [86,87]. Lupeol exhibited inhibitory potential on the AChE and BChE in vitro assays and Alzheimer’s model [72]. The findings of this research validate the potential of lemongrass extracts for utilization as ingredients in cosmetic skincare products either incorporated in antiaging, whitening or hypopigmenting preparations. Consequently, lemongrass emerges as a promising natural reservoir of easily accessible and cost-effective extracts abundant in antioxidant and enzyme inhibitory agents.

### 3.5. Molecular modelling

**Fig 3:**
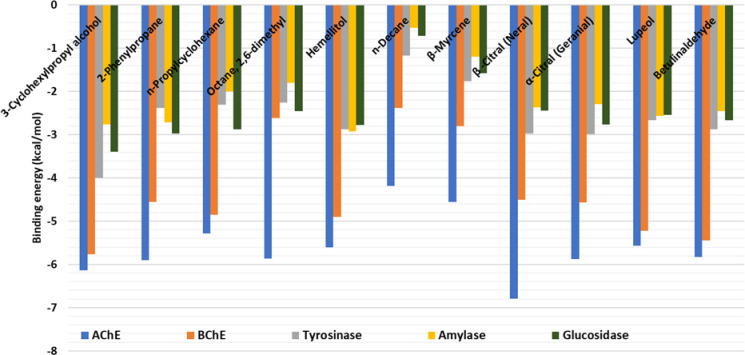
Docking score of main phytochemicals in the Lemongrass volatile oil and *n*-hexane extract.

**Fig 4:**
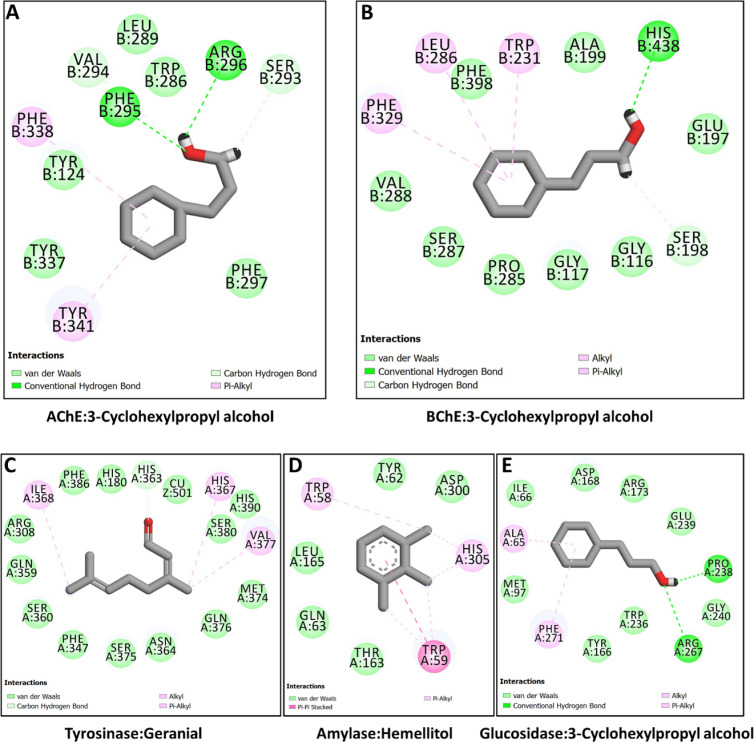
Protein-ligand interaction:(A) AChE and 3-Cyclohexylpropyl alcohol, (B) BChE and 3-Cyclohexylpropyl alcohol, (C) tyrosinase and geranial, (D) amylase and hemellitol, and (E) glucosidase and 3-Cyclohexylpropyl alcohol.

Molecular docking simulation was used to investigate the binding of bioactive components in lemongrass volatile oil and *n*-hexane extract. The docking binding energy scores are shown in (Fig 3). The dominating compounds exhibited promising binding affinity towards all the target proteins, with a particular affinity for AChE and BChE. For example, 3-cyclohexylpropyl alcohol bound strongly to both AChE (Fig 4A) and BChE (Fig 4B) via H-bonds, hydrophobic contacts, and a few van der Waals interactions with the enzymes’ active site residues.The compound formed a critical H-bond with Phe295 in the AChE active site (Fig 4A). Phe295 is a member acyl-binding pocket (Phe295, Phe297, and Trp236) responsible for substrate selectivity by restricting access to bigger members of the choline ester series and stabilizing ACh’s acetyl group [88]. Similarly, His438 formed an H-bond with cyclohexylpropyl alcohol, via the hydroxyl group, with BChE (Fig 4B). Because His438 is a part of the catalytic triad of BChE (Ser198, Glu325, and His438), this interaction can play a major role in blocking the enzyme’s activity [89].

Geranial also formed hydrophobic contacts but several van der Waals interactions with residues and catalytically Cu^2 +^ ion in the active site of tyrosinase (Fig 4C). Among these interactions, a van der with His363 and a hydrophobic contact His367 may contribute to the inhibition of the enzyme since both residues are members of the seven conserved histidine residues essential for tyrosinase activity [90]. The interaction of amylase enzyme with hemellitol hydrophobic contacts and a few van der Waals interactions buried the ligand in the enzyme’s catalytic pocket (Fig 4D). Most of these interacting residues, including Trp58 and Trp59 are also present in the crystal complex of amylase with acarbose [91].

Finally, like in the cases of AChE and BChE, the key interactions between 3-cyclohexylpropyl alcohol and glucosidase were H-bonds via hydroxyl group of the ligand and hydrophobic contacts with hydrophobic amino acid residues in the active site channel of the enzyme (Fig 4E). Blocking these residues, including Pro238 and Arg267, via H-bonds, may lead to inhibition because they play a role in the creation of glucose-binding cavities [92]. Therefore, they key interactions by which these compounds may block the activity of the selected enzymes were H-bonds, hydrophobic contacts, and van der Waals interactions.

## 4. Conclusions

The GC/MS analysis of lemongrass extracts showed that the VO was dominated by oxygenated monoterpenes (84.95%), primarily *α*-citral (36.08%), *β*-citral (34.22%), and *β*-myrcene (13.84%), while the HE was dominated by aliphatic hydrocarbons (42.98%), triterpenoids (20.14%), and aromatic hydrocarbons (17.25%). Besides, the hexane extract revealed considerable amounts of phenolics 7.44 mg GAE/g and flavonoids 1.91 mg RE/g. In the DPPH, ABTS, CUPRAC, FRAP and PM assays, the VO of lemongrass was more active and potent than the HE. In the metal chelating assay (MCA), the VO and HE showed comparable results of 31.16 and 25.76 mg EDTAE/g, respectively. Furthermore, for the AChE, BChE, and tyrosinase inhibitory activities, the VO showed more efficacy. That might be attributed to its high content of monoterpenes. While the hexane extract showed antidiabetic activity through inhibition of *α*-amylase and *α*-glucosidase enzymes. Molecular docking simulation was also used to study Lemongrass bioactive components and their binding sites to understand the mechanism. This study can assist in demonstrating the chemical composition and biological significance of lemongrass VO and HE. Additional *in-vivo* investigations are recommended for developing new candidates for oxidative stress diseases and diabetes.

## Supporting information

S1 File All experimental information regarding the determination of total phenolic and flavonoid content and determination of Antioxidant and Enzyme Inhibitory Effects can be found in the Supplementary Materials. (DOCX)

## References

[1] Gurib-Fakim A. Medicinal plants: traditions of yesterday and drugs of tomorrow. Mol Aspects Med. 2006;27(1):1–93. doi: 10.1016/j.mam.2005.07.008 16105678

[2] Zengin G, Yagi S, Eldahshan OA, Singab AN, Selvi S, Rodrigues MJ, et al. Decoding chemical profiles and biological activities of aerial parts and roots of *Eryngium thorifolium* Boiss by HPLC-MS/MS, GC-MS and *in vitro* chemical assays. Food Biosci. 2024;61:104556. doi: 10.1016/j.fbio.2024.104556

[3] Joos S, Glassen K, Musselmann B. Herbal medicine in primary healthcare in Germany: the Patient’s perspective. Evid-Based Complement Alternat Med. 2012;2012:294638. doi: 10.1155/2012/294638 23346197 PMC3549419

[4] Aly SH, Elissawy AM, Eldahshan OA, Elshanawany MA, Efferth T, Singab ANB. The pharmacology of the genus *Sophora* (Fabaceae): An updated review. Phytomedicine. 2019;64:153070. doi: 10.1016/j.phymed.2019.153070 31514082

[5] Mocan A, Carradori S, Locatelli M, Secci D, Cesa S, Mollica A, et al. Bioactive isoflavones from *Pueraria lobata* root and starch: Different extraction techniques and carbonic anhydrase inhibition. Food Chem Toxicol. 2018;112:441–7. doi: 10.1016/j.fct.2017.08.009 28807875

[6] Nagulapalli Venkata KC, Swaroop A, Bagchi D, Bishayee A. A small plant with big benefits: Fenugreek (*Trigonella foenum-graecum* Linn.) for disease prevention and health promotion. Mol Nutr Food Res. 2017;61(6):1600950. 10.1002/mnfr.201600950 28266134

[7] Balakrishnan B, Paramasivam S, Arulkumar A. Evaluation of the lemongrass plant (*Cymbopogon citratus*) extracted in different solvents for antioxidant and antibacterial activity against human pathogens. Asian Pac J Trop Dis. 2014;4:S134–9. doi: 10.1016/s2222-1808(14)60428-x

[8] Elgindi MR, El-Nassar Singab AB, Aly SH, Mahmoud II, Mohamed Elgindi CR. Phytochemical investigation and antioxidant activity of *Hyophorbe verschaffeltii* (Arecaceae). J Pharmacogn Phytochem. 2016;5:39–46.

[9] Aly SH, Elgindi MR, Singab AENB, Mahmoud II. *Hyophorbe verschaffeltii* DNA profiling, chemical composition of the lipophilic fraction, antimicrobial, anti-inflammatory and cytotoxic activities. Res J Pharm Biol Chem Sci. 2016;7:120–30.

[10] Tibenda JJ, Yi Q, Wang X, Zhao Q. Review of phytomedicine, phytochemistry, ethnopharmacology, toxicology, and pharmacological activities of *Cymbopogon* genus. Front Pharmacol. 2022;13:1–22. doi: 10.3389/fphar.2022.997918 PMC9465289 36105217

[11] Kim C, Park J, Lee H, Hwang DY, Park SH, Lee H. Evaluation of the EtOAc extract of lemongrass (*Cymbopogon citratus*) as a potential skincare cosmetic material for acne vulgaris. J Microbiol Biotechnol. 2022;32(5):594–601. doi: 10.4014/jmb.2201.01037 35484970 PMC9628876

[12] Avoseh O, Oyedeji O, Rungqu P, Nkeh-Chungag B, Oyedeji A. *Cymbopogon* species; ethnopharmacology, phytochemistry and the pharmacological importance. Molecules. 2015;20(5):7438–53. doi: 10.3390/molecules20057438 25915460 PMC6272507

[13] Ming LC, Figueiredo RO, Machado SR, Andrade RMC. Yield of essential oil of and citral content in different parts of lemongrass leaves (*Cymbopogon citratus* (dc) stape) Poaceae. Int Symp Med Aromatic Plants. 1995;426:555–9.

[14] Cheel J, Theoduloz C, Rodríguez J, Schmeda-Hirschmann G. Free radical scavengers and antioxidants from Lemongrass (*Cymbopogon citratus* (DC.) Stapf.). J Agric Food Chem. 2005;53(7):2511–7. doi: 10.1021/jf0479766 15796587

[15] Francisco V, Figueirinha A, Costa G, Liberal J, Lopes MC, García-Rodríguez C, et al. Chemical characterization and anti-inflammatory activity of luteolin glycosides isolated from lemongrass. J Funct Foods. 2014;10:436–43. doi: 10.1016/j.jff.2014.07.003

[16] Rhimi W, Mohammed MA, Zarea AAK, Greco G, Tempesta M, Otranto D, et al. Antifungal, antioxidant and Antibiofilm activities of essential oils of *Cymbopogon* spp. Antibiotics. 2022;11(6):829–13. doi: 10.3390/antibiotics11060829 35740234 PMC9220269

[17] Kpoviessi S, Bero J, Agbani P, Gbaguidi F, Kpadonou-Kpoviessi B, Sinsin B, et al. Chemical composition, cytotoxicity and in vitro antitrypanosomal and antiplasmodial activity of the essential oils of four *Cymbopogon* species from Benin. J Ethnopharmacol. 2014;151(1):652–9. doi: 10.1016/j.jep.2013.11.027 24269775

[18] Francisco V, Figueirinha A, Neves BM, García-Rodríguez C, Lopes MC, Cruz MT, et al. *Cymbopogon citratus* as source of new and safe anti-inflammatory drugs: bio-guided assay using lipopolysaccharide-stimulated macrophages. J Ethnopharmacol. 2011;133(2):818–27. doi: 10.1016/j.jep.2010.11.018 21075192

[19] Bharti SK, Kumar A, Prakash O, Krishnan S, Gupta AK. Essential oil of *Cymbopogon citratus* against diabetes: Validation by. vivo; 2013.

[20] Wang H, Zhang R, Zhang K, Chen X, Zhang Y. Antioxidant, hypoglycemic and molecular docking studies of Methanolic extract, fractions and isolated compounds from aerial parts of *Cymbopogon citratus* (DC.) Stapf. Molecules. 2022;27(9):2858. doi: 10.3390/molecules27092858 35566208 PMC9104508

[21] Zhao J, Fan Y, Cheng Z, Kennelly EJ, Long C. Ethnobotanical uses, phytochemistry and bioactivities of Cymbopogon plants: a review. J Ethnopharmacol. 2024;330:118181. doi: 10.1016/j.jep.2024.118181 38608798

[22] Cusumano G, Flores GA, Cetiz MV, Kurt U, Ak G, Saka E, et al. Small steps to the big picture for health‐promoting applications through the use of chickweed (*Stellaria media*): *In Vitro*, *In Silico*, and pharmacological network approaches. Food Sci Nutr. 2024;12(11):9295–313. doi: 10.1002/fsn3.4505 39620022 PMC11606822

[23] Aly SH, Mahmoud AMA, Abdel Mageed SS, Khaleel EF, Badi RM, Elkaeed EB, et al. Exploring the phytochemicals, antioxidant properties, and hepatoprotective potential of *Moricandia sinaica* leaves against paracetamol-induced toxicity: Biological evaluations and in Silico insights. PLoS One. 2024;19(10):e0307901. doi: 10.1371/journal.pone.0307901 39383154 PMC11463746

[24] Yerlikaya S, Zengin G, Mollica A, Baloglu MC, Celik Altunoglu Y, Aktumsek A. A multidirectional perspective for novel functional products: in vitro pharmacological activities and *in silico* studies on *Ononis natrix* subsp. hispanica. Front Pharmacol. 2017;8:600. doi: 10.3389/fphar.2017.00600 28919860 PMC5585257

[25] Mahomoodally MF, Vlaisavljevic S, Berezni S, Abdallah HH, Zengin G, Atanasov AG, et al. *Lotus aegaeus* (Gris.) Boiss and *Iberis sempervirens* L.: Chemical fingerprints, antioxidant potential, and inhibition activities and docking on key enzymes linked to global health problems. Ind Crops Prod. 2018;120:271–8. doi: 10.1016/j.indcrop.2018.04.056

[26] Abdel-Hameed UK, Abualghaith AS, Aly SH, Soliman MM, Munshi LA, Mohammed SAA, et al. GC/MS analysis and protective effects of *Mentha longifolia* L. essential oil against Antituberculosis drug-induced organs toxicity in Wistar albino rats. Plants (Basel, Switzerland). 2024;13(22):3231. doi: 10.3390/plants13223231 39599440 PMC11598752

[27] Aly SH, Kandil NH, Hemdan RM, Kotb SS, Zaki SS, Abdelaziz OM, et al. GC/MS profiling of the essential oil and lipophilic extract of *Moricandia sinaica* Boiss. and evaluation of their cytotoxic and antioxidant activities. Molecules. 2023;28(5):2193. doi: 10.3390/molecules28052193 36903440 PMC10004251

[28] Aly SH, Eldahshan OA, Al-Rashood ST, Binjubair FA, El Hassab MA, Eldehna WM, et al. Chemical constituents, antioxidant, and enzyme inhibitory activities supported by *In-Silico* study of *n*-Hexane extract and essential oil of guava leaves. Molecules. 2022;27(24):8979. doi: 10.3390/molecules27248979 36558111 PMC9781903

[29] Aly SH, El-Hassab MA, Elhady SS, Gad HA. Comparative Metabolic Study of *Tamarindus indica* L.’s Various Organs Based on GC/MS Analysis, *In Silico* and *In Vitro* Anti-Inflammatory and Wound Healing Activities. Plants. 2023;12(1):87. doi: 10.3390/plants12010087 PMC9824397 36616217

[30] Adams RP. Identification of essential oil components by gas chromatography/mass spectroscopy. ed. Illinois, USA: Allured Publishing Corporation; 2007. doi: 10.1016/0305-1978(96)83708-2

[31] Hussiny S, Elissawy A, Eldahshan O, Elshanawany M, Singab A-N. Phytochemical investigation using GC/MS analysis and evaluation of antimicrobial and cytotoxic activities of the lipoidal matter of leaves of *Sophora secundiflora* and *Sophora tomentosa*. Arch Pharm Sci Ain Shams Univ. 2020;4:207–14.

[32] Aly SH, Elissawy AM, Eldahshan OA, Elshanawany MA, Singab ANB. Variability of the chemical composition of the essential oils of flowers and the alkaloid contents of leaves of *Sophora secundiflora* and *Sophora tomentosa*. J Essent Oil Bear Plants. 2020;442–52. doi: 10.1080/0972060X.2020.1750489

[33] Fahmy NM, Fayez S, Uba AI, Shariati MA, Aljohani ASM, El-Ashmawy IM, et al. Comparative GC-MS analysis of fresh and dried curcuma essential oils with insights into their antioxidant and enzyme inhibitory activities. Plants. 2023;12(9):1785. doi: 10.3390/plants12091785 37176843 PMC10180709

[34] Abdelbaset S, El-Kersh DM, Ayoub IM, Eldahshan OA. GC-MS profiling of *Vitex pinnata* bark lipophilic extract and screening of its anti-TB and cytotoxic activities. Nat Prod Res. 2023;37(16):2718–24. doi: 10.1080/14786419.2022.2124512 36110061

[35] Zengin G, Aktumsek A. Investigation of antioxidant potentials of solvent extracts from different anatomical parts of *Asphodeline anatolica* E. Tuzlaci: an endemic plant to Turkey. Afr J Tradit Complement Altern Med. 2014;11(2):481–8. doi: 10.4314/ajtcam.v11i2.37 25435637 PMC4202661

[36] Grochowski DM, Uysal S, Aktumsek A, Granica S, Zengin G, Ceylan R, et al. In vitro enzyme inhibitory properties, antioxidant activities, and phytochemical profile of *Potentilla thuringiaca*. Phytochem Lett. 2017;20:365–72. doi: 10.1016/j.phytol.2017.03.005

[37] Uysal S, Zengin G, Locatelli M, Bahadori MB, Mocan A, Bellagamba G, et al. Cytotoxic and enzyme inhibitory potential of two *potentilla* species (*P. speciosa* L. and *P. reptans* Willd.) and their chemical composition. Front Pharmacol. 2017;8:1–11. doi: 10.3389/fphar.2017.00290 28588492 PMC5441381

[38] Fayez S, Fahmy NM, Zengin G, Yagi S, Uba AI, Eldahshan OA, et al. LC-MS/MS and GC-MS profiling, antioxidant, enzyme inhibition, and antiproliferative activities of *Thymus leucostomus* H ausskn. & V elen. extracts. Arch Pharm (Weinheim). 2023;356(12):e2300444. doi: 10.1002/ardp.202300444 37754205

[39] Noorgaldi S, Sarkala HB, Enayati A, Khori V, Zengin G, Jahanshahi M. Neuroprotective effect of *Potentilla reptans* L. root in the rat brain global ischemia/reperfusion model. Arch Pharm (Weinheim). 2023;356(11):e2300363. doi: 10.1002/ardp.202300363 37642540

[40] Kurumbail RG, Stevens AM, Gierse JK, McDonald JJ, Stegeman RA, Pak JY, et al. Structural basis for selective inhibition of cyclooxygenase-2 by anti-inflammatory agents. Nature. 1996;384(6610):644–8. doi: 10.1038/384644a0 8967954

[41] Maurus R, Begum A, Williams LK, Fredriksen JR, Zhang R, Withers SG, et al. Alternative catalytic anions differentially modulate human α-amylase activity and specificity. Biochemistry. 2008;47(11):3332–44. doi: 10.1021/bi701652t 18284212

[42] Gerlits O, Ho K-Y, Cheng X, Blumenthal D, Taylor P, Kovalevsky A, et al. A new crystal form of human acetylcholinesterase for exploratory room-temperature crystallography studies. Chem Biol Interact. 2019;309:108698. doi: 10.1016/j.cbi.2019.06.011 31176713 PMC6679772

[43] Rosenberry TL, Brazzolotto X, MacDonald IR, Wandhammer M, Trovaslet-Leroy M, Darvesh S, et al. Comparison of the binding of reversible inhibitors to human butyrylcholinesterase and acetylcholinesterase: a crystallographic, kinetic and calorimetric study. Molecules. 2017;22(12):2098. doi: 10.3390/molecules22122098 29186056 PMC6149722

[44] Ielo L, Deri B, Germano MP, Vittorio S, Mirabile S, Gitto R, et al. Exploiting the 1-(4-fluorobenzyl) piperazine fragment for the development of novel tyrosinase inhibitors as anti-melanogenic agents: Design, synthesis, structural insights and biological profile. Eur J Med Chem. 2019;178:380–9. doi: 10.1016/j.ejmech.2019.06.019 31202126

[45] Karade SS, Hill ML, Kiappes JL, Manne R, Aakula B, Zitzmann N, et al. N-substituted Valiolamine derivatives as potent inhibitors of endoplasmic reticulum α-glucosidases I and II with antiviral activity. J Med Chem. 2021;64(24):18010–24. doi: 10.1021/acs.jmedchem.1c01377 34870992

[46] Zengin G, Dall’Acqua S, Sinan KI, Uba AI, Sut S, Peron G, et al. Gathering scientific evidence for a new bioactive natural ingredient: the combination between chemical profiles and biological activities of *Flueggea virosa* extracts. Food Biosci. 2022;49:101967. doi: 10.1016/j.fbio.2022.101967

[47] Kurt-Celep I, Zheleva-Dimitrova D, Gevrenova R, Uba AI, Zengin G, Yıldıztugay E, et al. An in-depth study on the metabolite profile and biological properties of *Primula auriculata* extracts: a fascinating sparkle on the way from nature to functional applications. Antioxidants (Basel, Switzerland). 2022;11(7):1377. doi: 10.3390/antiox11071377 35883868 PMC9312287

[48] Pettersen EF, Goddard TD, Huang CC, Couch GS, Greenblatt DM, Meng EC, et al. UCSF Chimera—a visualization system for exploratory research and analysis. J Comput Chem. 2004;25(13):1605–12. doi: 10.1002/jcc.20084 15264254

[49] Morris GM, Huey R, Lindstrom W, Sanner MF, Belew RK, Goodsell DS, et al. AutoDock4 and AutoDockTools4: automated docking with selective receptor flexibility. J Comput Chem. 2009;30(16):2785–91. doi: 10.1002/jcc.21256 19399780 PMC2760638

[50] Świątek L, Sieniawska E, Sinan KI, Zengin G, Uba AI, Bene K, et al. Bridging the chemical profiles and biological effects of *Spathodea campanulat* a extracts: a new contribution on the road from natural treasure to pharmacy shelves. Molecules. 2022;27(15):4694. doi: 10.3390/molecules27154694 35897865 PMC9330408

[51] Elebeedy D, Ghanem A, Aly SH, Ali MA, Faraag AHI, El-Ashrey MK, et al. Synergistic antiviral activity of *Lactobacillus acidophilus* and *Glycyrrhiza glabra* against Herpes Simplex-1 Virus (HSV-1) and Vesicular Stomatitis Virus (VSV): experimental and *In Silico* insights. BMC Microbiol. 2023;23(1):173. doi: 10.1186/s12866-023-02911-z 37391715 PMC10311774

[52] El-Nashar HAS, Aly SH, Hritcu L, Eldahshan OA. Mango (*Mangifera indica* L.): phytochemical profile, nutritional aspects and potential anti-diabetic therapeutics studies. In: Ancient and traditional foods, plants, herbs and spices used in diabetes. CRC Press; 2024. p. 279–295.

[53] Aly SH, Elhawary EA, Elissawy AM, Mostafa NM, Eldahshan OA, Singab ANB. 13 Brown Algae (Phaeophyta). Aquatic medicinal plants. CRC Press; 2023. 24 p.

[54] Madi YF, Choucry MA, Meselhy MR, El-Kashoury E-SA. Essential oil of *Cymbopogon citratus* cultivated in Egypt: seasonal variation in chemical composition and anticholinesterase activity. Nat Prod Res. 2021;35(21):4063–7. doi: 10.1080/14786419.2020.1713125 31960718

[55] Moustafa MAM, Awad M, Amer A, Hassan NN, Ibrahim EDS, Ali HM, et al. Correction: Insecticidal activity of lemongrass essential oil as an eco-friendly agent against the black cutworm *Agrotis ipsilon* (lepidoptera: Noctuidae), (Insects (2021), 12, 737, 10.3390/insects12080737). Insects. 2021;12:1–12. doi: 10.3390/insects12110991 PMC8396863 34442303

[56] Dangol S, Poudel DK, Ojha PK, Maharjan S, Poudel A, Satyal R, et al. Essential oil composition analysis of *Cymbopogon* species from Eastern Nepal by GC-MS and chiral GC-MS, and antimicrobial activity of some major compounds. Molecules. 2023;28(2):543. doi: 10.3390/molecules28020543 36677603 PMC9863348

[57] Mohamad S, Ismail NN, Parumasivam T, Ibrahim P, Osman H, A. Wahab H. Antituberculosis activity, phytochemical identification of *Costus speciosus* (J. Koenig) Sm., *Cymbopogon citratus* (DC. Ex Nees) Stapf., and *Tabernaemontana coronaria* (L.) Willd. and their effects on the growth kinetics and cellular integrity of Mycobacteri. BMC Complement Altern Med. 2018;18(1):1–14. doi: 10.1186/s12906-017-2077-5 29310671 PMC5759295

[58] Cortes-Torres AG, López-Castillo GN, Marín-Torres JL, Portillo-Reyes R, Luna F, Baca BE, et al. *Cymbopogon citratus* essential oil: extraction, GC–MSPhytochemical analysis, antioxidant activity, and *In Silico* molecular docking for protein targets related to CNS. Curr Issues Mol Biol. 2023;45(6):5164–79. doi: 10.3390/cimb45060328 37367077 PMC10297282

[59] Guerrini A, Tacchini M, Chiocchio I, Grandini A, Radice M, Maresca I, et al. A comparative study on chemical compositions and biological activities of four *Amazonian Ecuador* essential oils: *Curcuma longa* L. (Zingiberaceae), *Cymbopogon citratus* (DC.) Stapf, (Poaceae), *Ocimum campechianum* Mill. (Lamiaceae), and Zingiber officinale R. Antibiotics. 2023;12(1):177. doi: 10.3390/antibiotics12010177 36671378 PMC9855031

[60] Aly SH, Elissawy AM, Mahmoud AMA, El-Tokhy FS, Mageed SSA, Almahli H, et al. Synergistic effect of *Sophora japonica* and *Glycyrrhiza glabra* flavonoid-rich fractions on wound healing: *In Vivo* and molecular docking studies. Molecules. 2023;28(7):2994. doi: 10.3390/molecules28072994 37049756 PMC10096052

[61] El-Nashar HAS, Aly SH, Ahmadi A, El-Shazly M. The impact of polyphenolics in the management of breast cancer: mechanistic aspects and recent patents. Recent Pat Anticancer Drug Discov. 2021;17:358–79. doi: 10.2174/1574892816666211213090623 34961465

[62] Saber FR, Aly SH, Khallaf MA, El-Nashar HAS, Fahmy NM, El-Shazly M, et al. *Hyphaene thebaica* (Areceaeae) as a promising functional food: extraction, analytical techniques, bioactivity, food, and industrial applications. Food Anal Methods. 2022;16(9-10):1447–67. doi: 10.1007/s12161-022-02412-1

[63] Aly SH, Elissawy AM, Salah D, Alfuhaid NA, Zyaan OH, Mohamed HI, et al. Phytochemical investigation of three *Cystoseira* species and their Larvicidal activity supported with In Silico studies. Mar Drugs. 2023;21(2):117. doi: 10.3390/md21020117 36827158 PMC9967941

[64] Aly SH, Elissawy AM, Allam AE, Farag SM, Eldahshan OA, Elshanawany MA, et al. New quinolizidine alkaloid and insecticidal activity of *Sophora secundiflora* and *Sophora tomentosa* against Culex pipiens (Diptera: Culicidae). Nat Prod Res. 2022;36(11):2722–34. doi: 10.1080/14786419.2021.1919108 33974474

[65] Sousa R, Figueirinha A, Batista MT, Pina ME. Formulation effects in the antioxidant activity of extract from the leaves of *Cymbopogon citratus* (Dc) stapf. Molecules. 2021;26(15):4518. doi: 10.3390/molecules26154518 34361669 PMC8348009

[66] Unuigbe C, Enahoro J, Erharuyi O, Okeri HA. Phytochemical analysis and antioxidant evaluation of lemon grass (*Cymbopogon citratus* DC.) Stapf leaves. J Appl Sci Environ Manag. 2019;23(2):223–8. doi: 10.4314/jasem.v23i2.4

[67] Sah SY, Sia CM, Chang SK, Ang YK, Yim HS. Antioxidant capacity and total phenolic content of lemon grass (*Cymbopogon citratus*) leave. Ann Food Sci Technol. 2012;13:150–5.

[68] Méabed EMH, Abou-Sreea AIB, Roby MHH. Chemical analysis and giardicidal effectiveness of the aqueous extract of *Cymbopogon citratus* Stapf. Parasitol Res. 2018;117(6):1745–55. doi: 10.1007/s00436-018-5855-1 29666923

[69] Li C-C, Yu H-F, Chang C-H, Liu Y-T, Yao H-T. Effects of lemongrass oil and citral on hepatic drug-metabolizing enzymes, oxidative stress, and acetaminophen toxicity in rats. J Food Drug Anal. 2018;26(1):432–8. doi: 10.1016/j.jfda.2017.01.008 29389585 PMC9332636

[70] Yang S-M, Hua K-F, Lin Y-C, Chen A, Chang J-M, Kuoping Chao L, et al. Citral is renoprotective for focal segmental glomerulosclerosis by inhibiting oxidative stress and apoptosis and activating Nrf2 pathway in mice. PLoS One. 2013;8(9):e74871. doi: 10.1371/journal.pone.0074871 24069362 PMC3775727

[71] Gupta R, Sharma AK, Sharma MC, Dobhal MP, Gupta RS. Evaluation of antidiabetic and antioxidant potential of lupeol in experimental hyperglycaemia. Nat Prod Res. 2012;26(12):1125–9. doi: 10.1080/14786419.2011.560845 22043924

[72] Park JS, Rehman IU, Choe K, Ahmad R, Lee HJ, Kim MO. A triterpenoid lupeol as an antioxidant and anti-neuroinflammatory agent: impacts on oxidative stress in Alzheimer’s disease. Nutrients. 2023;15(13):3059. doi: 10.3390/nu15133059 37447385 PMC10347110

[73] Santiago LA, Mayor ABR. Lupeol: an antioxidant triterpene in *Ficus pseudopalma* Blanco (Moraceae). Asian Pac J Trop Biomed. 2014;4(2):109–18. doi: 10.1016/S2221-1691(14)60218-5 25182281 PMC3819478

[74] Zengin G, Mostafa NM, Abdelkhalek Y M, Eldahshan OA. Antioxidant and Enzyme Inhibitory Activities of Rhoifolin flavonoid: *In-vitro* and *In-Silico* Studies. Chem Biodivers. 2023:e202300117. 37498319 10.1002/cbdv.202300117

[75] Lee H-A, Kim M-J, Han J-S. Alleviating effects of lupeol on postprandial hyperglycemia in diabetic mice. Toxicol Res (Camb). 2021;10(3):495–500. doi: 10.1093/toxres/tfab019 34141163 PMC8201585

[76] Singh M, Kaur M, Kukreja H, Chugh R, Silakari O, Singh D. Acetylcholinesterase inhibitors as Alzheimer therapy: from nerve toxins to neuroprotection. Eur J Med Chem. 2013;70:165–88. doi: 10.1016/j.ejmech.2013.09.050 24148993

[77] Stanciu GD, Luca A, Rusu RN, Bild V, Beschea Chiriac SI, Solcan C, et al. Alzheimer’s disease pharmacotherapy in relation to cholinergic system involvement. Biomolecules. 2019;10(1):40. doi: 10.3390/biom10010040 31888102 PMC7022522

[78] Aly SH, Elissawy AM, Fayez AM, Eldahshan OA, Elshanawany MA, Singab ANB. Neuroprotective effects of *Sophora secundiflora*, *Sophora tomentosa* leaves and formononetin on scopolamine-induced dementia. Nat Prod Res. 2020;35(24):5848–52. doi: 10.1080/14786419.2020.1795853 32696670

[79] El-Nashar HAS, Eldehna WM, Al-Rashood ST, Alharbi A, Eskandrani RO, Aly SH. GC/ MS analysis of essential oil and enzyme inhibitory activities of *Syzygium cumini* (Pamposia) grown in docking studies. Molecules. 2021;26(22):6984. doi: 10.3390/molecules26226984 34834076 PMC8618078

[80] Ighodaro OM, Akinloye OA, Ugbaja RN, Omotainse SO, Faokunla O. FT-IR analysis of *Sapium ellipticum* (Hochst) pax ethanol leaf extract and its inhibitory effects on pancreatic α-amylase and intestinal α-glucosidase activities in vitro. Egypt J Basic Appl Sci. 2016;3(4):343–9. doi: 10.1016/j.ejbas.2016.09.003

[81] Boaduo NKK, Katerere D, Eloff JN, Naidoo V. Evaluation of six plant species used traditionally in the treatment and control of diabetes mellitus in South Africa using *in vitro* methods. Pharm Biol. 2014;52(6):756–61. doi: 10.3109/13880209.2013.869828 24559378

[82] Rolff M, Schottenheim J, Decker H, Tuczek F. Copper–O 2 reactivity of tyrosinase models towards external monophenolic substrates: molecular mechanism and comparison with the enzyme. Chem Soc Rev. 2011;40(7):4077–98. doi: 10.1039/c0cs00202j 21416076

[83] Korany DA, Nilofar N, Zengin G, Eldahshan OA. Chemical constituents, antioxidant, and enzyme inhibitory potentials supported by In‐Silico studies of the n‐hexane extract and essential oil of *Platycladus orientalis* (L.) franco leaves. Chem Biodivers. e202402000. 39462973 10.1002/cbdv.202402000

[84] Matsuura R, Ukeda H, Sawamura M. Tyrosinase inhibitory activity of citrus essential oils. J Agric Food Chem. 2006;54(6):2309–13. doi: 10.1021/jf051682i 16536612

[85] Manjia JN, Njoya EM, Harishchander A, Munvera AM, Ogundolie FA, Mkounga P, et al. Anti-elastase, Anti-tyrosinase, and anti-inflammatory activities of three compounds isolated from *Psorospermum aurantiacum*: *In Silico* and *In Vitro* assays. Rev Bras Farmacogn. 2024;34(5):1116–28. doi: 10.1007/s43450-024-00558-z

[86] Orhan I, Kartal M, Kan Y, Şener B. Activity of essential oils and individual components against acetyland butyrylcholinesterase. Z Naturforsch C. 2008;63(7-8):547–53. doi: 10.1515/znc-2008-7-813 18810999

[87] Oliveira ER de, Alves DS, Carvalho GA, Oliveira BMRG de, Aazza S, Bertolucci SKV. Toxicity of *Cymbopogon flexuosus* essential oil and citral for Spodoptera frugiperda. Ciênc Agrotecnologia. 2018;42:408–19.

[88] Hörnberg A, Tunemalm A-K, Ekström F. Crystal structures of acetylcholinesterase in complex with organophosphorus compounds suggest that the acyl pocket modulates the aging reaction by precluding the formation of the trigonal bipyramidal transition state. Biochemistry. 2007;46(16):4815–25. doi: 10.1021/bi0621361 17402711

[89] Chen X, Fang L, Liu J, Zhan C-G. Reaction pathway and free energy profiles for butyrylcholinesterase-catalyzed hydrolysis of acetylthiocholine. Biochemistry. 2012;51(6):1297–305. doi: 10.1021/bi201786s 22304234 PMC3292049

[90] Noh H, Lee SJ, Jo H-J, Choi HW, Hong S, Kong K-H. Histidine residues at the copper-binding site in human tyrosinase are essential for its catalytic activities. J Enzyme Inhib Med Chem. 2020;35(1):726–32. doi: 10.1080/14756366.2020.1740691 32180482 PMC7144311

[91] Nahoum V, Roux G, Anton V, Rougé P, Puigserver A, Bischoff H, et al. Crystal structures of human pancreatic α-amylase in complex with carbohydrate and proteinaceous inhibitors. Biochem J. 2000;346(1):201–8. doi: 10.1042/0264-6021:3460201 PMC1220841 10657258

[92] Barrett T, Suresh CG, Tolley SP, Dodson EJ, Hughes MA. The crystal structure of a cyanogenic β-glucosidase from white clover, a family 1 glycosyl hydrolase. Structure. 1995;3(9):951–60. doi: 10.1016/s0969-2126(01)00229-5 8535788

